# Supplementary material for: Oral administration of Blautia wexlerae ameliorates obesity and type 2 diabetes via metabolic remodeling of the gut microbiota
Source: Nat Commun. 2022 Aug 18;13:4477. doi: 10.1038/s41467-022-32015-7 (PMC9388534; doi:10.1038/s41467-022-32015-7)
Supplement: Supplementary file 1 — Supplementary Information [file 41467_2022_32015_MOESM1_ESM.pdf]

Supplementary Information: Oral administration of *Blautia wexlerae* ameliorates obesity and type 2 diabetes via metabolic remodeling of the gut microbiota

J. Kunisawa et al.

Supplementary Figure 1

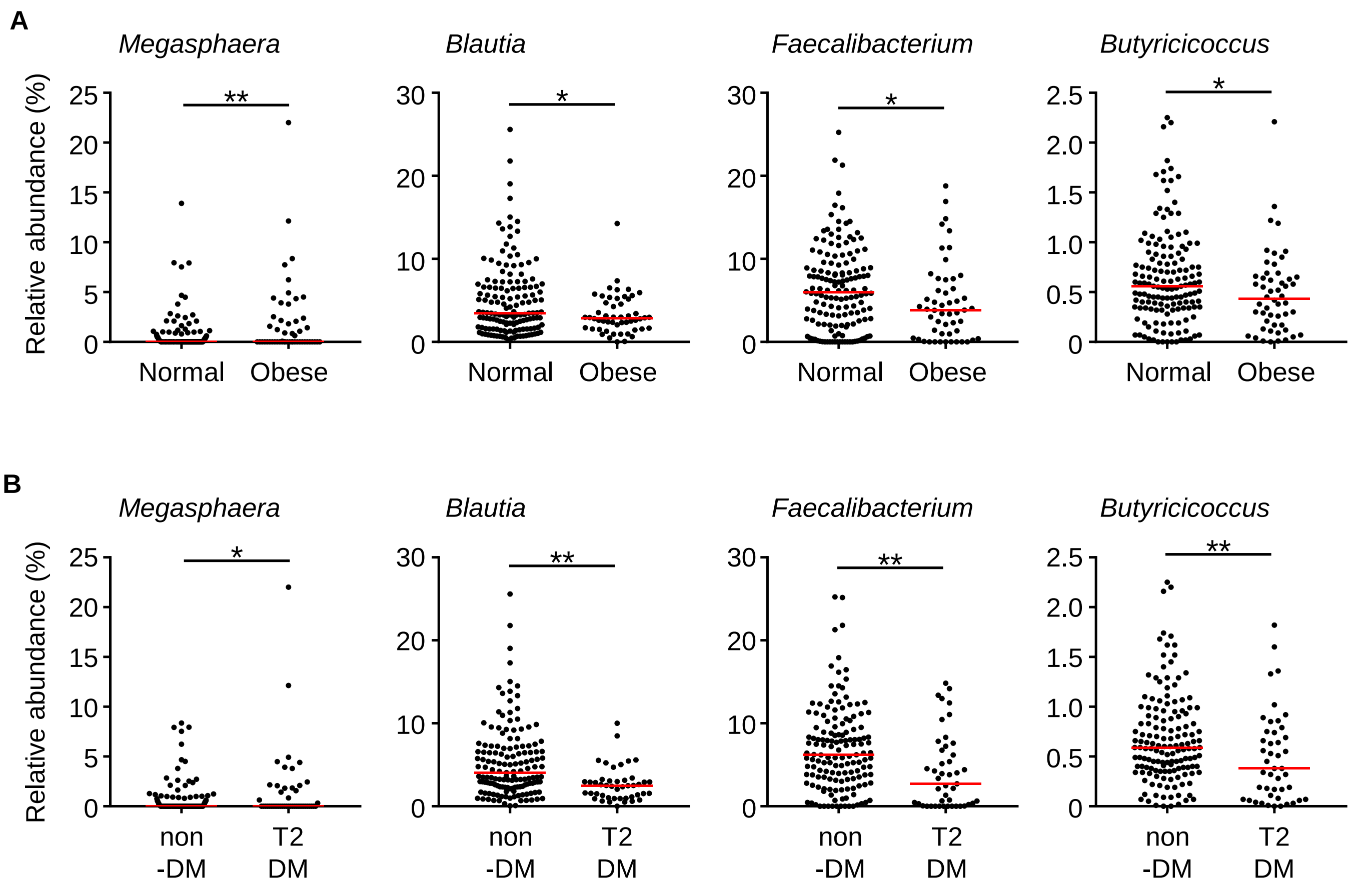

Supplementary Figure 1. Relative abundance of human intestinal bacteria
(A) Comparison of relative abundance of human intestinal bacteria between normal-weight (BMI 18.5–24.9, n = 148) and obese (BMI ≥ 25, n = 52) Japanese adult participants. (B) Comparison of relative abundance of human intestinal bacteria between non-diabetic subjects (non-DM, n = 147) and those with type 2 diabetes (T2DM, n = 45). Statistical significance was evaluated by using the two-tailed Mann–Whitney *U*-test; $*P < 0.05$; $**P < 0.01$. Red lines indicate mean.

Supplementary Figure 2

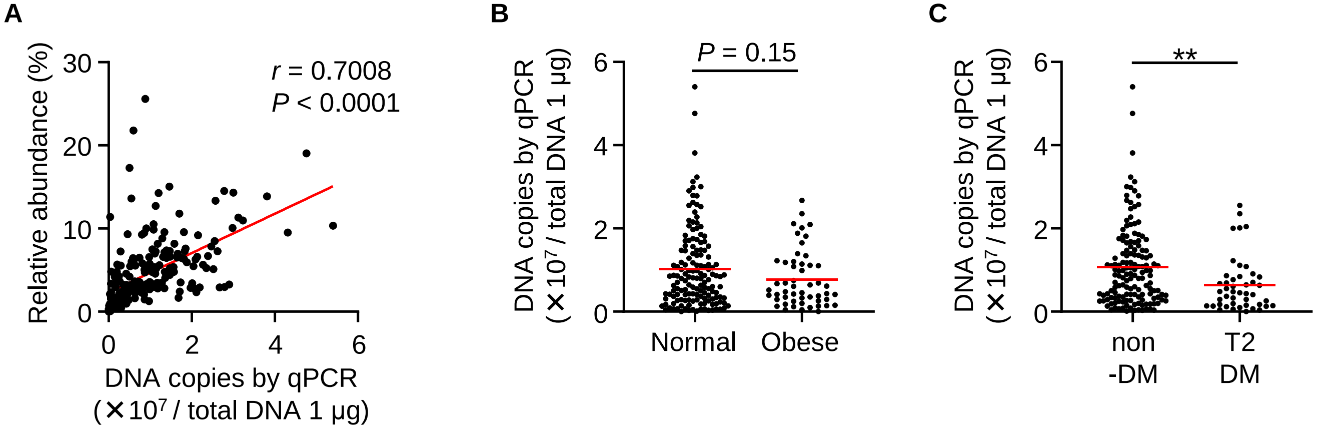

Supplementary Figure 2. Validation of *Blautia* abundance by quantitative PCR analysis
(A) Spearman correlation analysis between the relative abundance of the *Blautia* genus according to 16S rRNA gene amplicon sequencing analysis and the DNA copy number of the *Blautia* genus as measured through quantitative PCR analysis. (B) Comparison of the *Blautia* DNA copy number of human intestinal bacteria between normal-weight (BMI 18.5–24.9, n = 148) and obese (BMI ≥ 25, n = 52) Japanese adult participants. (C) Comparison of the *Blautia* DNA copy number of human intestinal bacteria between non-diabetic subjects (non-DM, n = 147) and those with type 2 diabetes (T2DM, n = 45). Statistical significance was evaluated by using the two-tailed Mann–Whitney *U*-test; $^{**}P = 0.0014$. Red lines indicate single linear regression (A) or mean (B, C).

Supplementary Figure 3

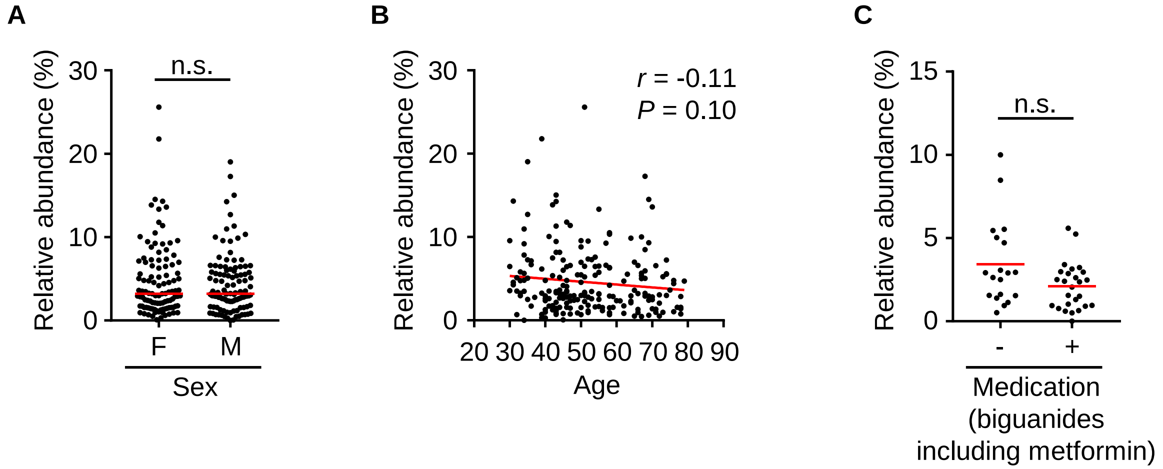

Supplementary Figure 3. Effects of potential confounding factors of sex, age, and medication
(A) Comparison of the abundance of the *Blautia* genus according to sex (F, female, n = 107; M, male, n = 110). (B) Pearson correlation analysis between *Blautia* genus abundance and age. (C) Comparison of *Blautia* genus abundance between T2DM patients non-treated (–) or treated (+) with biguanides, such as metformin. Statistical significance was evaluated by using the two-tailed Mann-Whitney *U*-test; n.s., not significant (A, C). Red lines indicate mean (A, C) or single linear regression (B).

Supplementary Figure 4

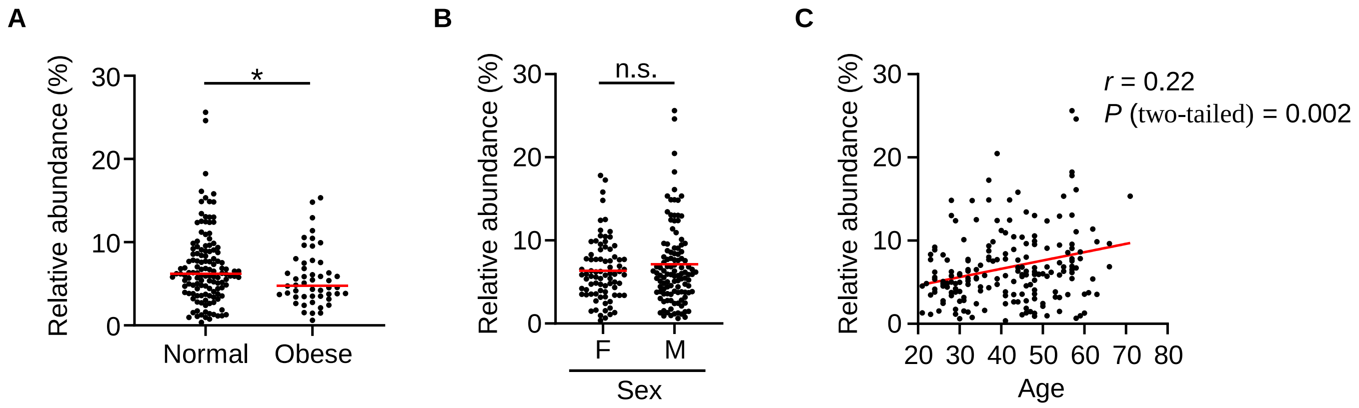

Supplementary Figure 4. Relative abundance of *Blautia* genus in validation cohort
(A) Comparison of relative abundance of *Blautia* genus between normal-weight (BMI 18.5–24.9, n = 132) and obese (BMI ≥ 25, n = 50) Japanese adult participants. (B) Comparison of the abundance of the *Blautia* genus according to sex (F, female, n = 81; M, male, n = 114). (C) Pearson correlation analysis between *Blautia* genus abundance and age (n = 195). Statistical significance was evaluated by using the two-tailed Mann–Whitney *U*-test; **P* = 0.0435; n.s., not significant (A, B). Red lines indicate mean (A, B) or single linear regression (C).

Supplementary Figure 5

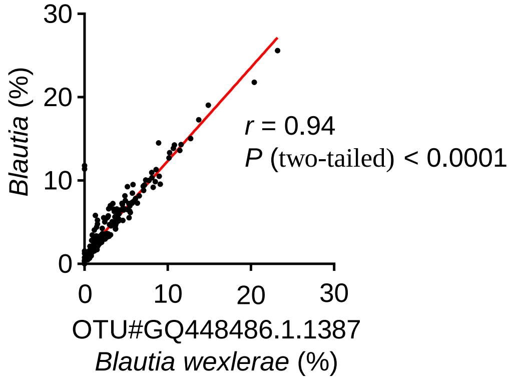

Supplementary Figure 5. Positive correlation (Pearson correlation analysis) between the abundance of *Blautia* genus and *B. wexlerae* (OTU GQ448486.1.1387)
Red line indicates single linear regression.

Supplementary Figure 6

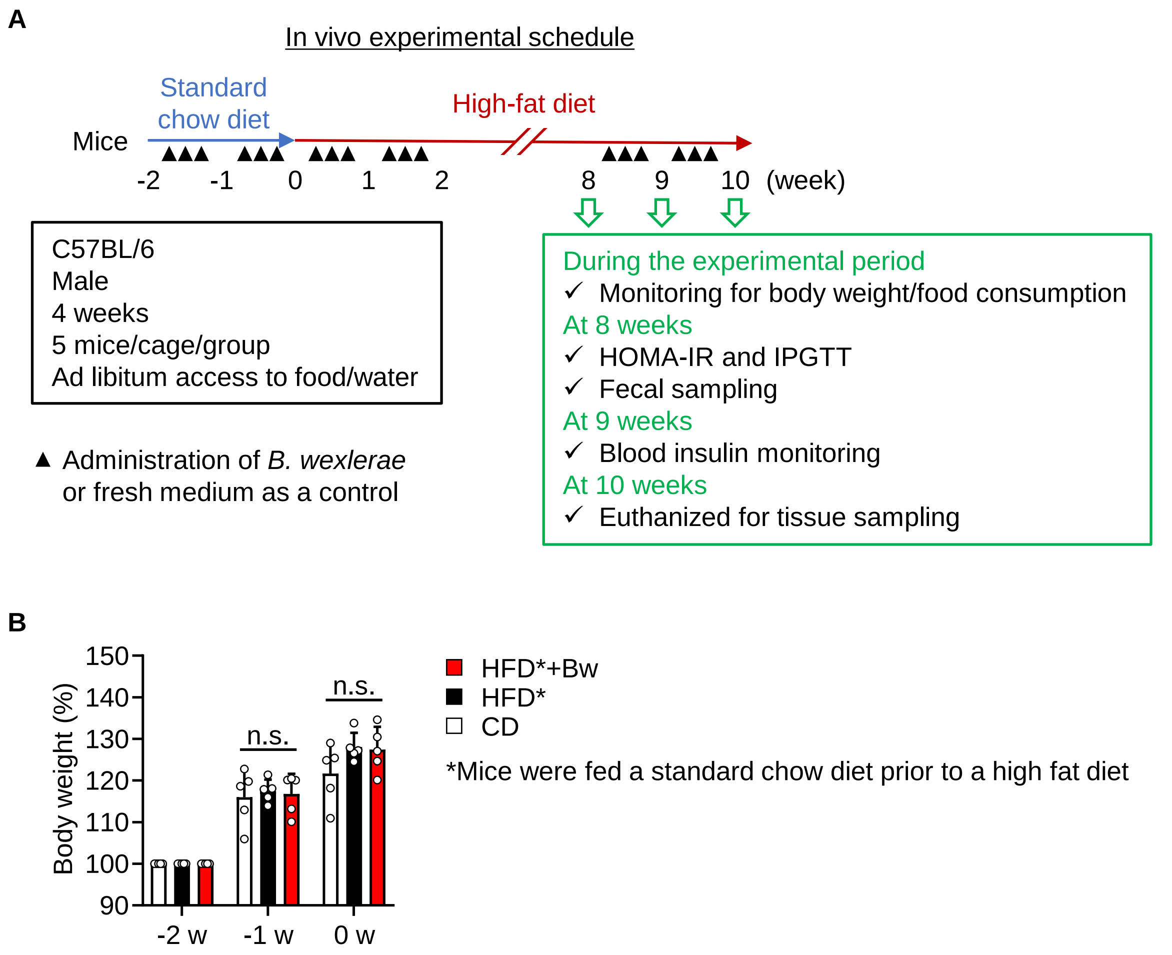

Supplementary Figure 6. Schedule for mouse experiment
(A) The schedule for investigating the effects of oral administration of *B. wexlerae* on high-fat diet (HFD)-induced obesity and diabetes in mice. (B) Mice were weighed weekly (n = 5, mean ± 1 SD) prior to feeding a high-fat diet. Statistical significance was evaluated by using one-way ANOVA; n.s., not significant. CD, CD-fed mice; HFD, HFD-fed mice; HFD+Bw, HFD-fed mice orally supplemented with *B. wexlerae*. Data are representative of 2 independent experiments.

Supplementary Figure 7

A

Composition of diet (ingredients, g %)

	Standard chow diet	High fat diet
Casein	14.0	25.0
L-Cystine	0.18	0.375
Corn Starch	46.5692	-
Pregelatinized Corn Starch	15.5	14.869
Sucrose	10.0	20.0
Soybean Oil	4.0	10.0
Beef tallow	-	5.0
Lard	-	15.0
Cellulose Powder	5.0	5.0
AIN-93 Mineral Mix	3.5	3.5
AIN-93 Vitamin Mix	1.0	1.0
Choline bitartrate	0.25	0.25
t-Butylhydroquinone	0.0008	0.006
Total	100.0	100.0
kcal/100 g	353.5	475.2

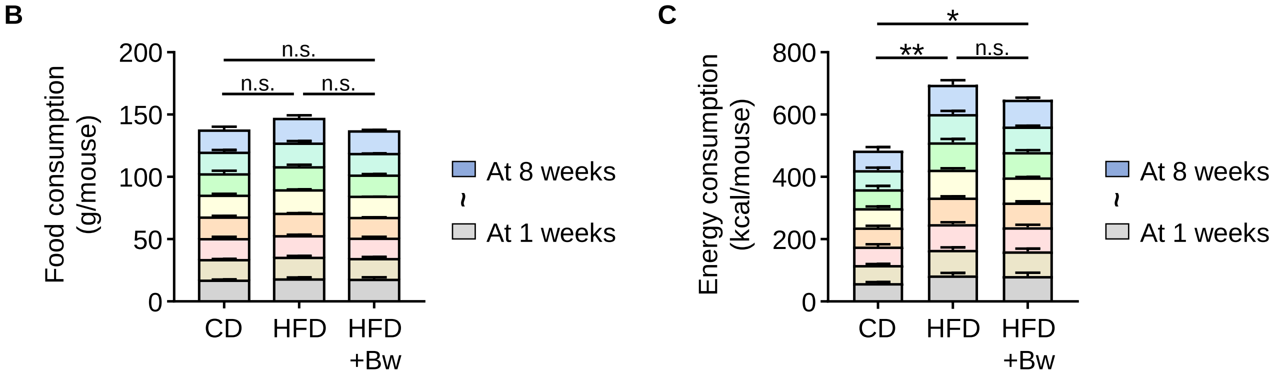

Supplementary Figure 7. Food intake of mice

(A) Composition of diet. (B) The amount of chow consumed (g) by each cage of 5 mice was measured each week in 3 independent experiments, and the average intake per mouse is shown (n = 3, mean ± 1 SD). (C) The calculated amount of energy consumed (kcal) (n = 3, mean ± 1 SD). Statistical significance was evaluated by using one-way ANOVA; *P = 0.0206; **P = 0.0063; n.s., not significant. CD, CD-fed mice; HFD, HFD-fed mice; HFD+Bw, HFD-fed mice orally supplemented with *B. wexlerae*.

Supplementary Figure 8

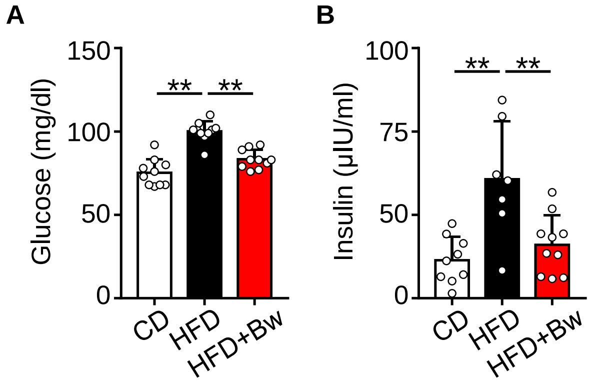

Supplementary Figure 8. Blood diabetes indicators in mice

(A) Fasting blood glucose. Data are combined from 2 independent experiments (n = 10, mean ± 1 SD). (B) Fasting blood insulin. Data are combined from 2 independent experiments withouthemolytic samples (n = 7–10, mean ± 1 SD). Statistical significance was evaluated by using one-way ANOVA; $^{**}P < 0.01$. CD, CD-fed mice; HFD, HFD-fed mice; HFD+Bw, HFD-fed mice orally supplemented with *B. wexlerae*.

Supplementary Figure 9

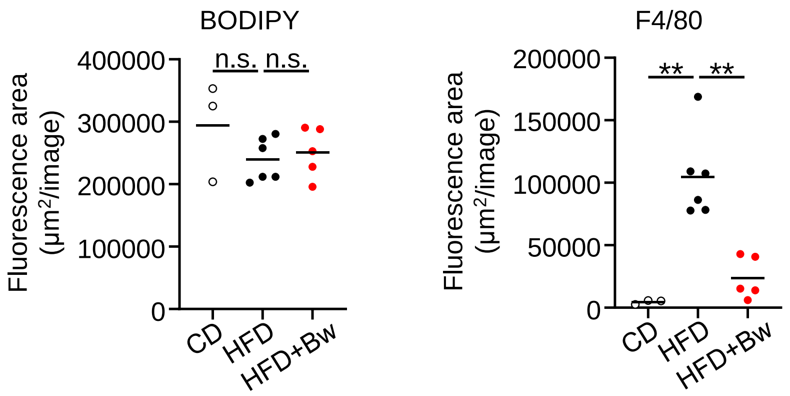

Figure S9. Fluorescence area for BODIPY and F4/80 in eAT sections
CD, CD-fed mice; HFD, HFD-fed mice; HFD+Bw, HFD-fed mice orally supplemented with *B. wexlerae*. Statistical significance was evaluated by using one-way ANOVA; $^{**}P < 0.01$; n.s., not significant. Data are combined from 2 independent experiments (n = 3–6, mean ± 1 SD).

Supplementary Figure 10

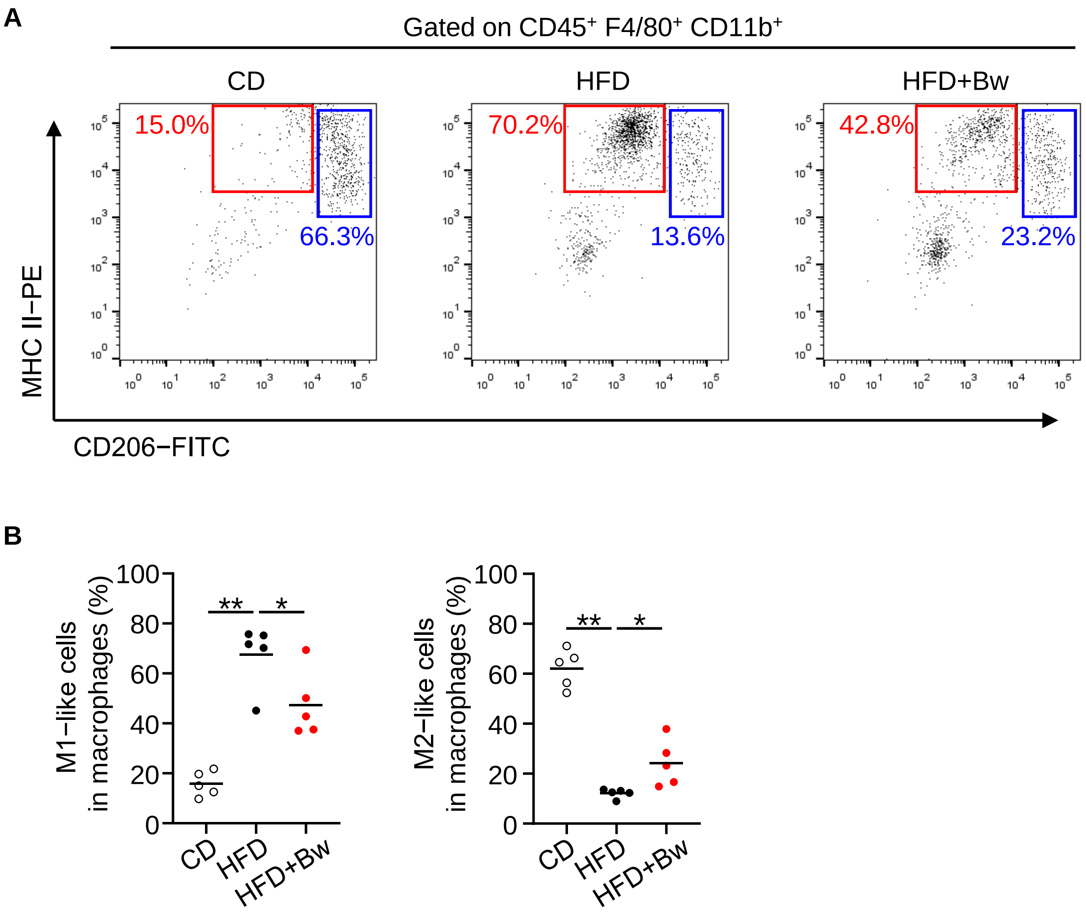

Supplementary Figure 10. Flow cytometric analysis of M1- and M2-like macrophages in eAT
(A) Representative flow cytometry plot. (B) The percentage of M1/M2-like macrophages. The $CD45^{+}CD11b^{+}F4/80^{+}$ cells were defined as the macrophage population. Among macrophages, the MHC $II^{+/high}CD206^{-/low}$ cells and MHC $II^{+}CD206^{high}$ cells were defined as the M1- and M2-like macrophage population, respectively. CD, CD-fed mice; HFD, HFD-fed mice; HFD+Bw, HFD-fed mice orally supplemented with *B. wexlerae*. Statistical significance was evaluated by using one-way ANOVA; *$P < 0.05$; **$P < 0.01$; n.s., not significant. Data are representative of 2 independent experiments (n = 5, mean).

Supplementary Figure 11

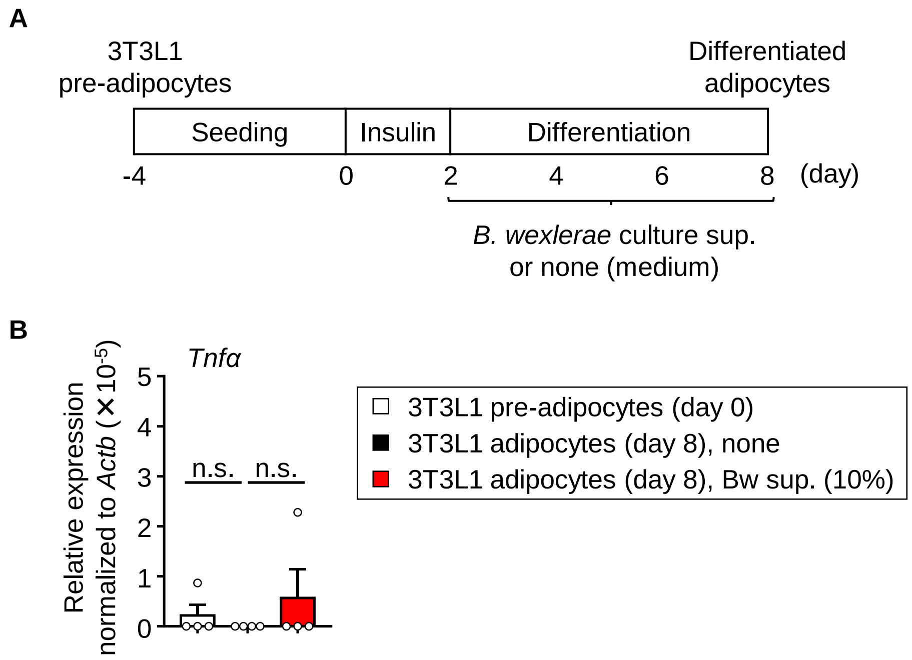

Supplementary Figure 11. Culture of 3T3L1 adipocytes
(A) The procedure for differentiating 3T3L1 pre-adipocytes into mature adipocytes. (B) Gene expression of *Tnfα*, an inflammatory cytokine, in 3T3L1 pre-adipocytes and 3T3L1 adipocytes treated without (none) or with the supernatant (sup.) from *B. wexlerae* cultures at a final concentration of 10%. Statistical significance was evaluated by using one-way ANOVA; n.s., not significant. Data are representative of 2 independent experiments (n = 4, mean ± 1 SD).

Supplementary Figure 12

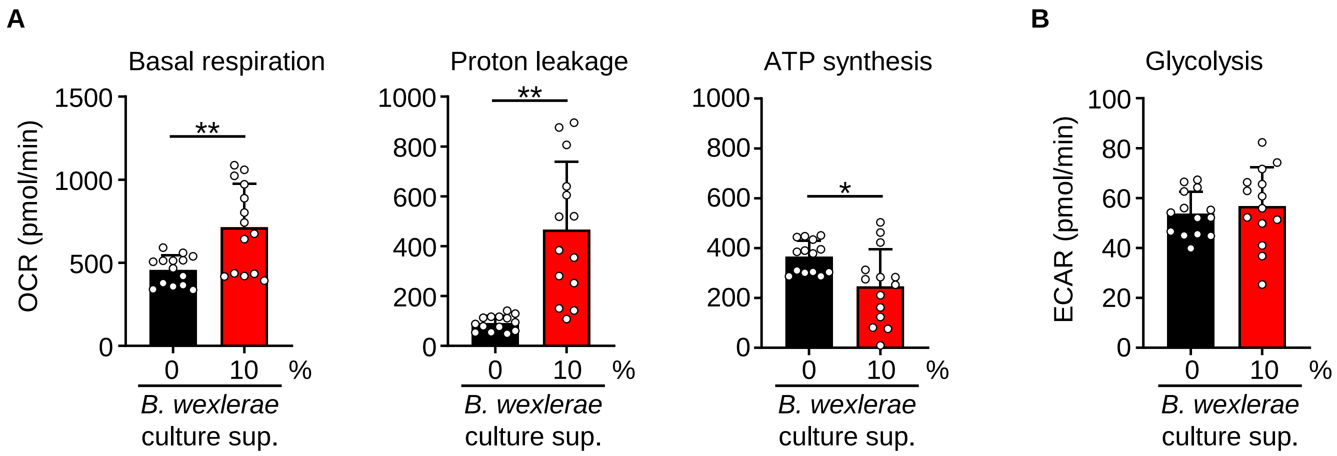

Supplementary Figure 12. Measurement of oxygen consumption rate and extracellular acidification rate in 3T3L1 adipocytes
(A) Oxygen consumption rate (OCR) and (B) extracellular acidification rate (ECAR) were measured in 3T3L1 adipocytes treated without or with the supernatant from *B. wexlerae* cultures at a final concentration of 10% by using an XF24 extracellular flux analyzer. OCR for basal respiration, proton leakage, and ATP synthesis were measured by using an XF Mito Stress Kit. Statistical significance was evaluated by using one-way ANOVA; **P* < 0.05; ***P* < 0.01. Data are combined from 2 independent experiments (n = 14, mean ± 1 SD).

Supplementary Figure 13

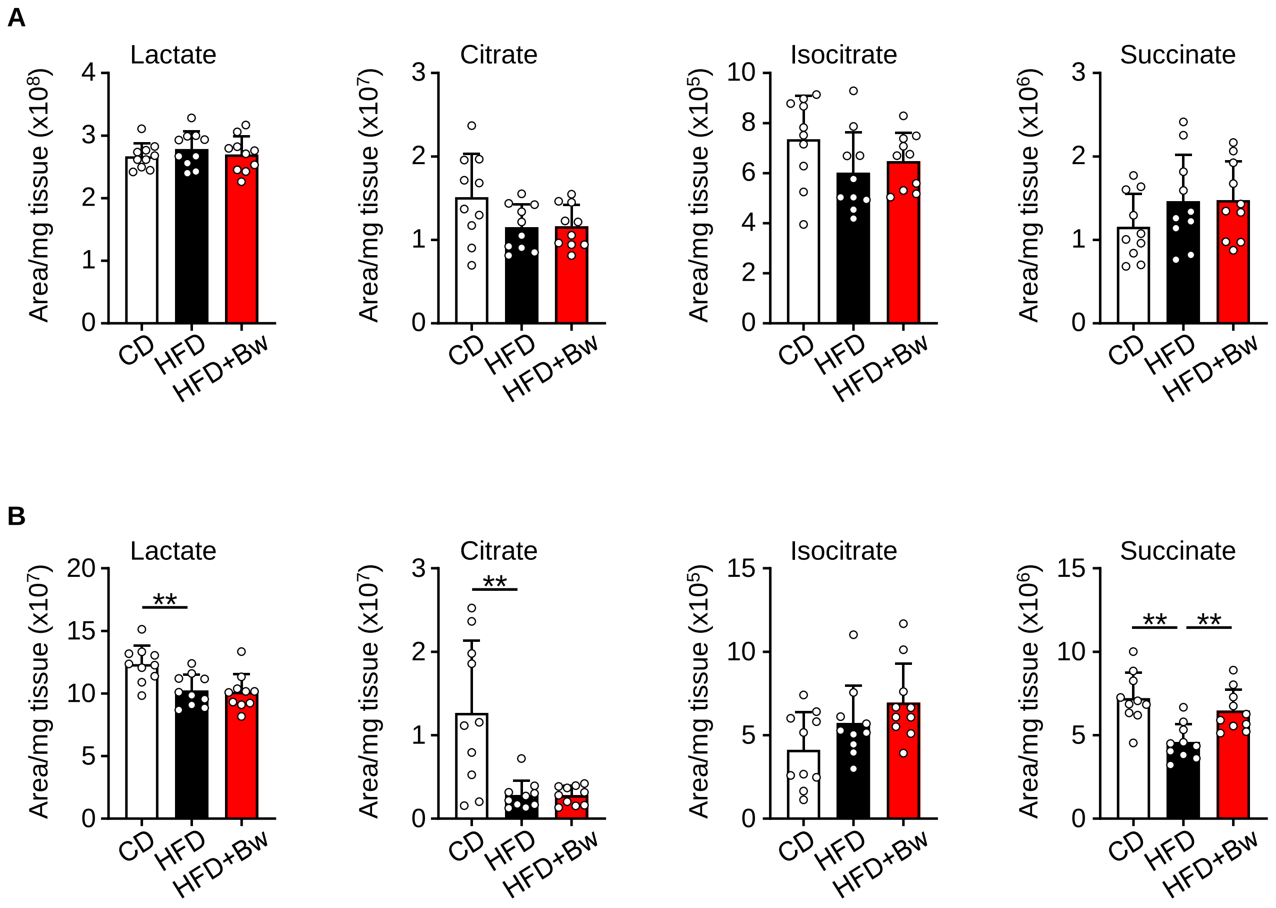

Supplementary Figure 13. Energy metabolism in gastrocnemius muscle and liver of mice
Representative metabolites of glycolysis (lactate) and the TCA cycle (citrate, isocitrate, and succinate) in the gastrocnemius muscle (A) and liver (B) of mice were measured by using liquid chromatography-tandem mass spectroscopy (LC-MS/MS). Statistical significance was evaluated by using one-way ANOVA; *$P < 0.05$; **$P < 0.01$; n.s., not significant. CD, CD-fed mice; HFD, HFD-fed mice; HFD+Bw, HFD-fed mice orally supplemented with *B. wexlerae*. Data are combined from 2 independent experiments (n = 10, mean ± 1 SD).

Supplementary Figure 14

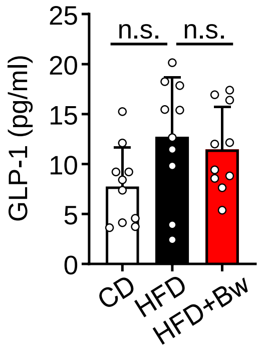

Supplementary Figure 14. Serum GLP-1 in mice

Statistical significance was evaluated by using one-way ANOVA; n.s., not significant. CD, CD-fed mice; HFD, HFD-fed mice; HFD+Bw, HFD-fed mice orally supplemented with *B. wexlerae*. Data are combined from 2 independent experiments (n = 10, mean ± 1 SD).

Supplementary Figure 15

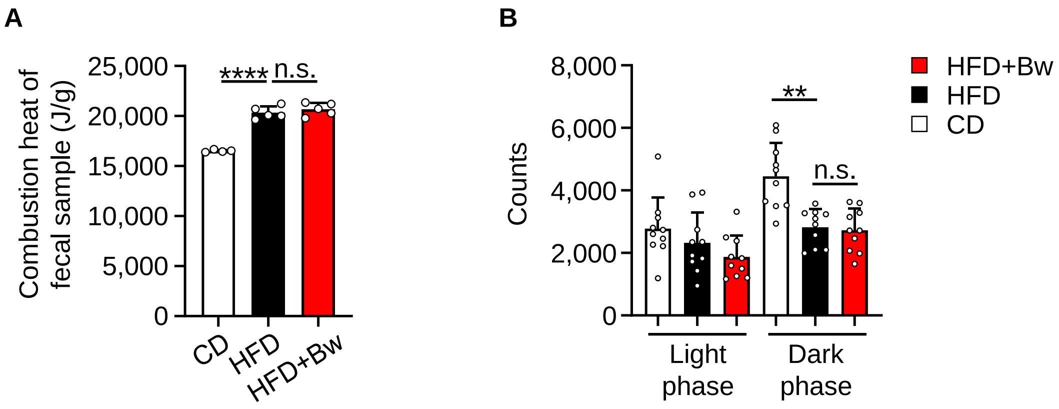

Supplementary Figure 15. Energy excretion in mice

(A) Energy values for mouse feces. Data are representative of 2 independent experiments (n = 4–5, mean ± 1 SD). (B) Spontaneous activity of mice during 24 h. Data are combined from 2 independent experiments (n = 10, mean ± 1 SD). Statistical significance was evaluated by using one-way ANOVA; ****P < 0.0001; **P = 0.0010; n.s., not significant. CD, CD-fed mice; HFD, HFD-fed mice; HFD+Bw, HFD-fed mice orally supplemented with *B. wexlerae*.

Supplementary Figure 16

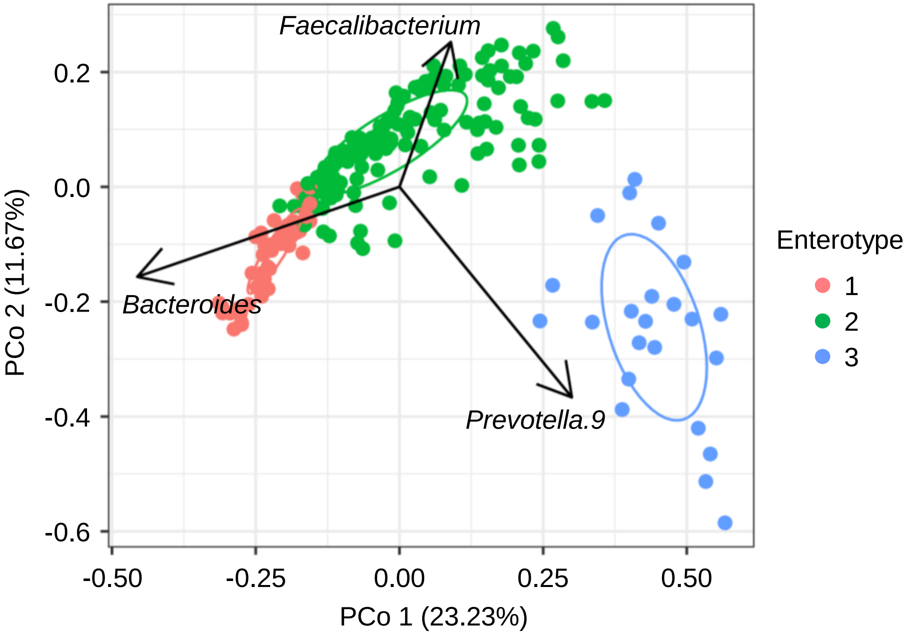

Supplementary Figure 16. Distribution of human gut microbiome according to principal coordinates analysis (PCoA, genus-level Jensen-Shannon Divergence)
1, *Bacteroides* enterotype (red); 2, *Faecalibacterium* enterotype (green); 3, *Prevotella* enterotype (blue). Arrows, enterotype drivers.

Supplementary Figure 17

Comparison of *Blautia* and *Bacteroides*

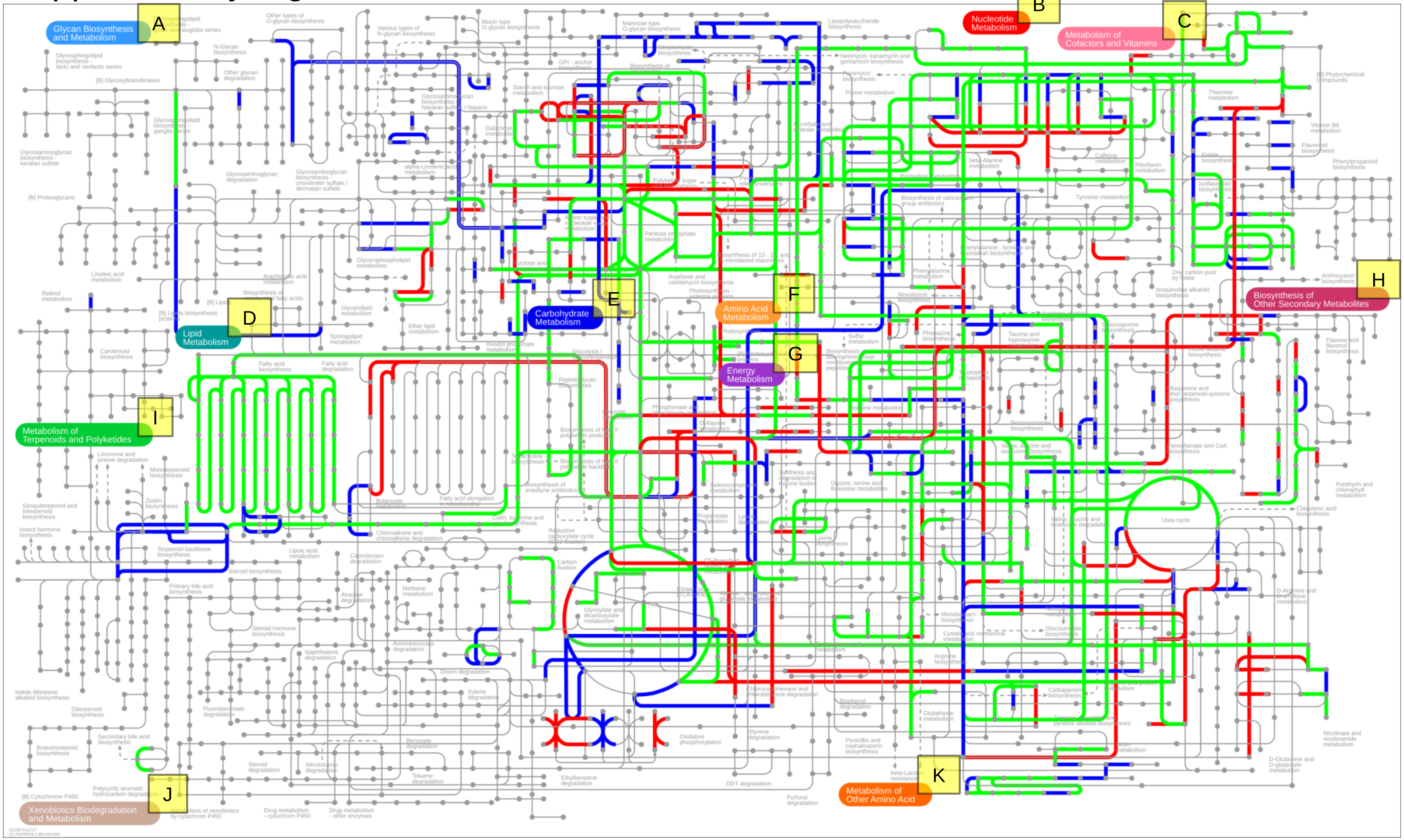

A	Glycan Biosynthesis and Metabolism	E	Carbohydrate Metabolism	I	Metabolism of Terpenoids and Polyketides
B	Nucleotide Metabolism	F	Amino Acid Metabolism	J	Xenobiotics Biodegradation and Metabolism
C	Metabolism of Cofactors and Vitamins	G	Energy Metabolism	K	Metabolism of Other Amino Acid
D	Lipid Metabolism	H	Biosynthesis of Other Secondary Metabolites		

— *Blautia* — *Bacteroides* — In common

Supplementary Figure 17. *In silico* analysis of metabolic pathways according to the presence of KEGG orthologous groups compared between *Blautia* (KEGG organism code: rob) and *Bacteroides* (KEGG organism code: bvu) by using iPATH3.0.

Supplementary Figure 18

Comparison of *Blautia* and *Prevotella*

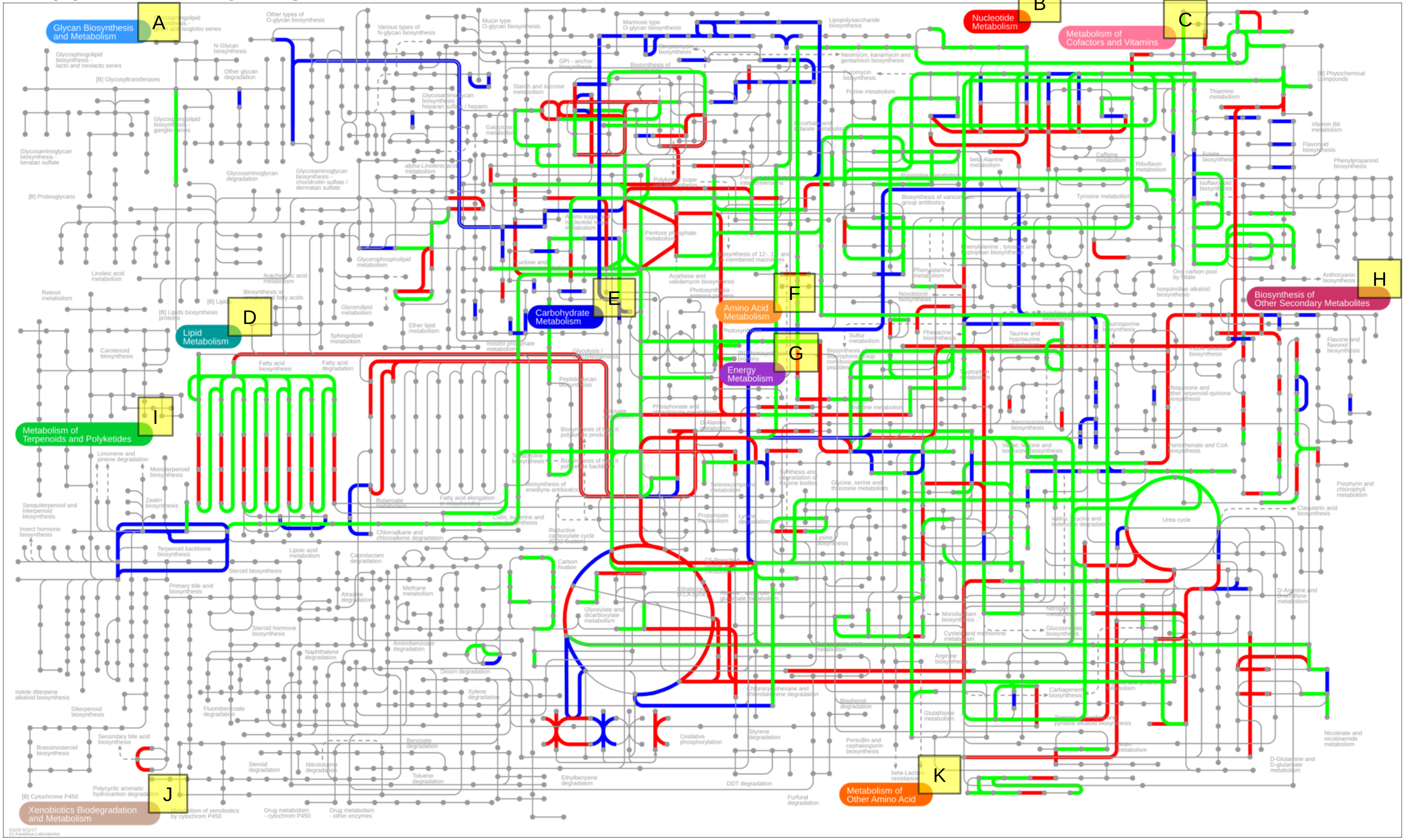

A	Glycan Biosynthesis and Metabolism	E	Carbohydrate Metabolism	I	Metabolism of Terpenoids and Polyketides
B	Nucleotide Metabolism	F	Amino Acid Metabolism	J	Xenobiotics Biodegradation and Metabolism
C	Metabolism of Cofactors and Vitamins	G	Energy Metabolism	K	Metabolism of Other Amino Acid
D	Lipid Metabolism	H	Biosynthesis of Other Secondary Metabolites		

Blautia — *Prevotella* — In common

Supplementary Figure 18. *In silico* analysis of metabolic pathways according to the presence of KEGG orthologous groups compared between *Blautia* (KEGG organism code: rob) and *Prevotella* (KEGG organism code: pru) by using iPATH3.0.

Supplementary Figure 19

Comparison of *Blautia* and *Faecalibacterium*

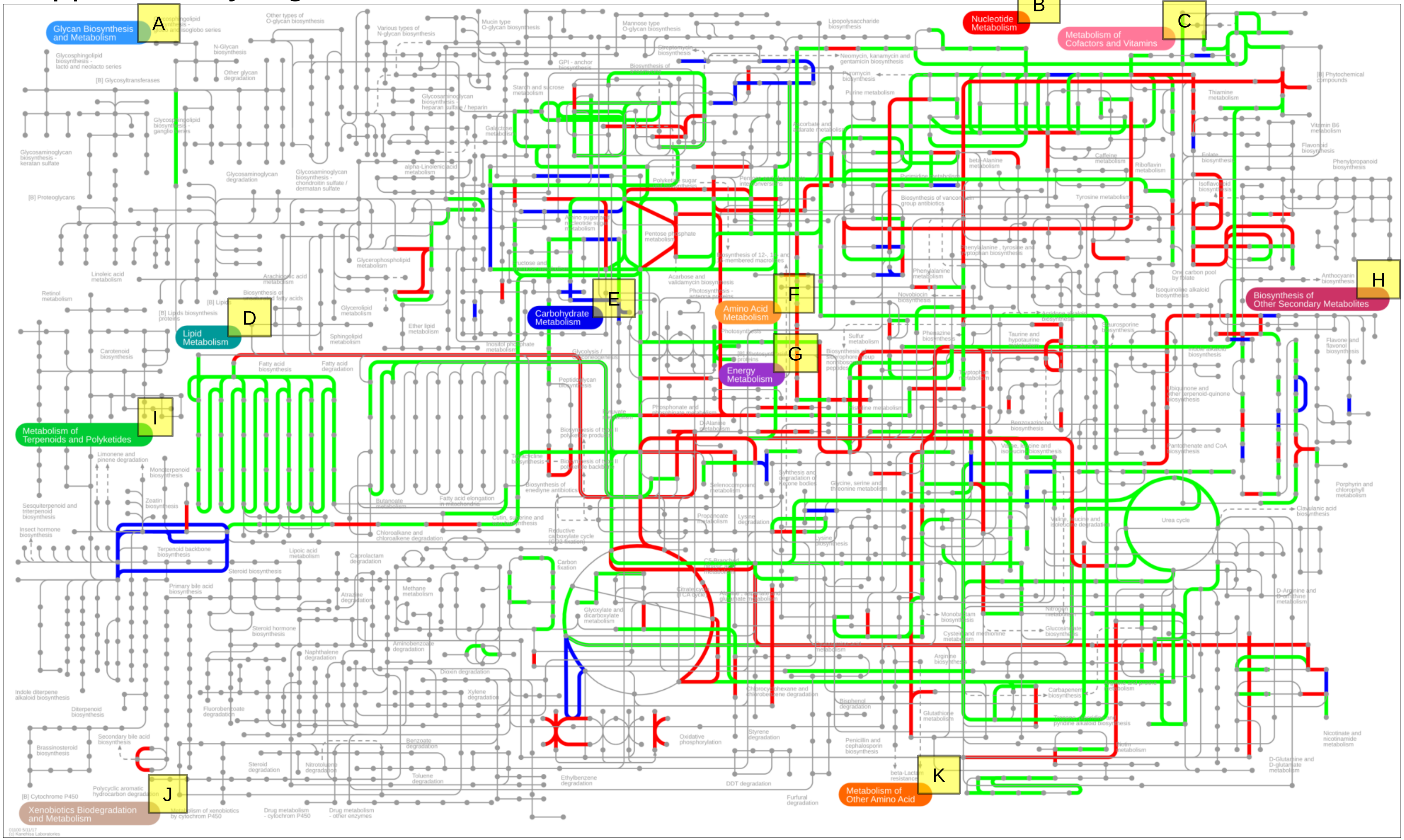

A	Glycan Biosynthesis and Metabolism
B	Nucleotide Metabolism
C	Metabolism of Cofactors and Vitamins
D	Lipid Metabolism
E	Carbohydrate Metabolism
F	Amino Acid Metabolism
G	Energy Metabolism
H	Biosynthesis of Other Secondary Metabolites
I	Metabolism of Terpenoids and Polyketides
J	Xenobiotics Biodegradation and Metabolism
K	Metabolism of Other Amino Acid

— *Blautia* — *Faecalibacterium* — In common

Supplementary Figure 19. *In silico* analysis of metabolic pathways according to the presence of KEGG orthologous groups compared between *Blautia* (KEGG organism code: rob) and *Faecalibacterium* (KEGG organism code: fpr) by using iPATH3.0.

Supplementary Figure 20

KEGG orthologous groups unique to *Blautia*

K00016, K00087, K00111, K00128, K00194, K00197, K00198, K00335, K00336, K00394, K00395, K00853, K00917, K00926, K01026, K01214, K01239, K01464, K01487, K01494, K01512, K01626, K01628, K01693, K01698, K01749, K01758, K01845, K02083, K02119, K02122, K02160, K02189, K02203, K02304, K02433, K02434, K02435, K02492, K02502, K02588, K02773, K03060, K03621, K04072, K05305, K05350, K05878, K05879, K06042, K06209, K06928, K07404, K08093, K08094, K08744, K13479, K13542, K13954, K14260, K15023

Identification of enriched pathways by TargetMine (Benjamini Hochberg)

Metabolic pathways
Microbial metabolism in diverse environments
Porphyrin and chlorophyll metabolism
Biosynthesis of amino acids
Carbon metabolism
Pyruvate metabolism
Biosynthesis of secondary metabolites
Chloroalkane and chloroalkene degradation
Purine metabolism
Carbon fixation pathways in prokaryotes

Supplementary Figure 20. Pathways uniquely enriched in *Blautia*.
61 KEGG orthologous groups were uniquely identified in *Blautia* but not in *Bacteroides*, *Prevotella*, or *Faecalibacterium* by using iPATH3.0. TargetMine showed 10 metabolic pathways enriched in the 61 KEGG orthologous groups ($P < 0.01$, Benjamini–Hochberg analysis).

Supplementary Figure 21

Unique metabolic pathways of *Blautia*

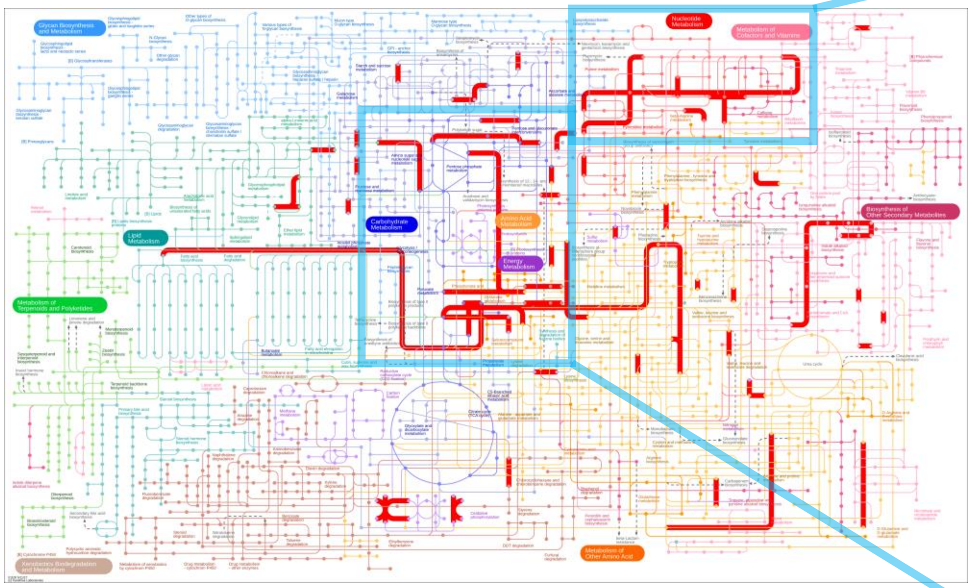

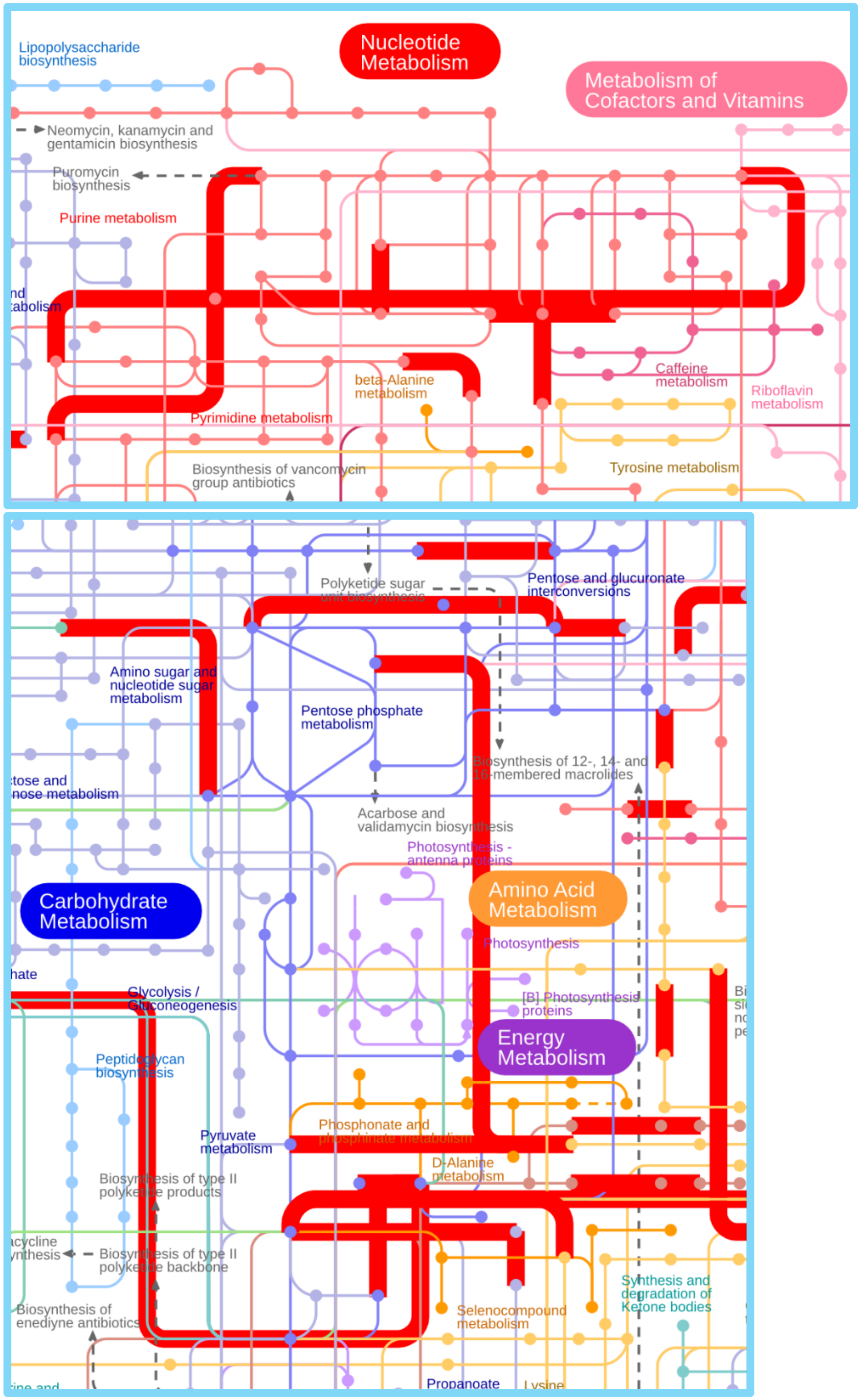

Supplementary Figure 21. Unique metabolic pathways (red lines) of *Blautia*.

Supplementary Figure 22

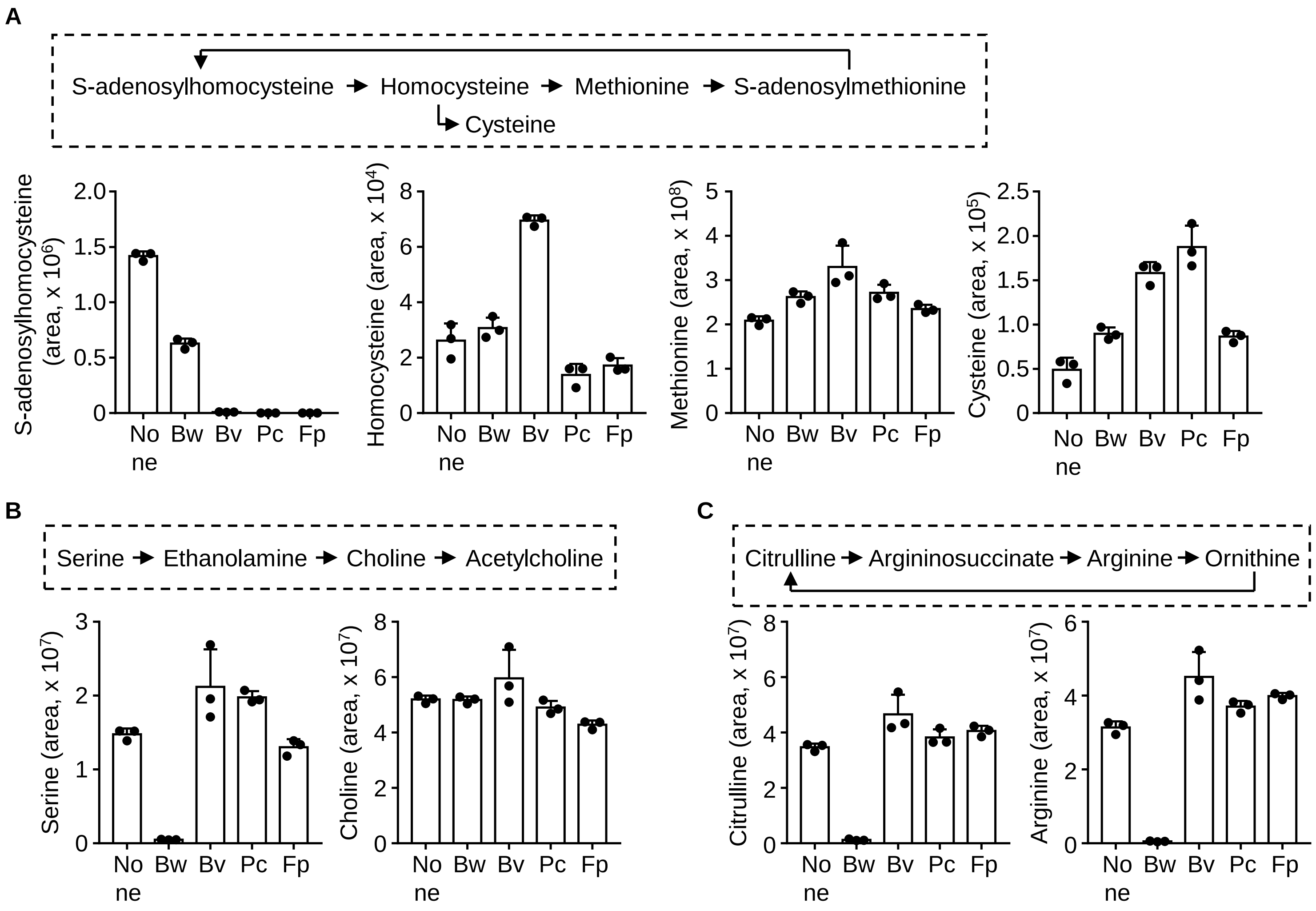

Supplementary Figure 22. Relative abundance of bacterial metabolites in amino acid metabolism
Schematic diagrams of synthetic pathways for (A) S-adenosylmethionine, (B) acetylcholine, and (C) L-ornithine and related metabolites measured by LC-MS/MS in fresh medium (none) and the supernatant from cultures of *B. wexlerae* (Bw) and major intestinal bacteria-cultured supernatant including *Bacteroides vulgatus* (Bv), *Prevotella copri* (Pc), and *Faecalibacterium prausnitzii* (Fp). Data are representative of 2 independent experiments (n = 3, mean ± 1 SD).

Supplementary Figure 23

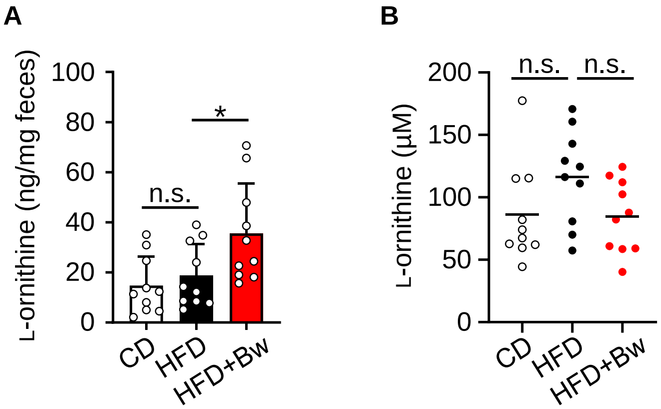

Supplementary Figure 23. Quantitative measurement by LC-MS/MS of L-ornithine in mice
(A) Mouse fecal sample was collected from mice at 8 weeks, stored at –80° C, and analyzed by LC-MS/MS. (B) Mouse serum was collected at 10 weeks when they were euthanized for tissue sampling, stored at –80° C, and analyzed by LC-MS/MS. Statistical significance was evaluated by using one-way ANOVA; **P* = 0.0497; n.s., not significant. CD, CD-fed mice; HFD, HFD-fed mice; HFD+Bw, HFD-fed mice orally supplemented with *B. wexlerae*. Data are combined from 2 independent experiments (n = 10, mean ± 1 SD).

Supplementary Figure 24

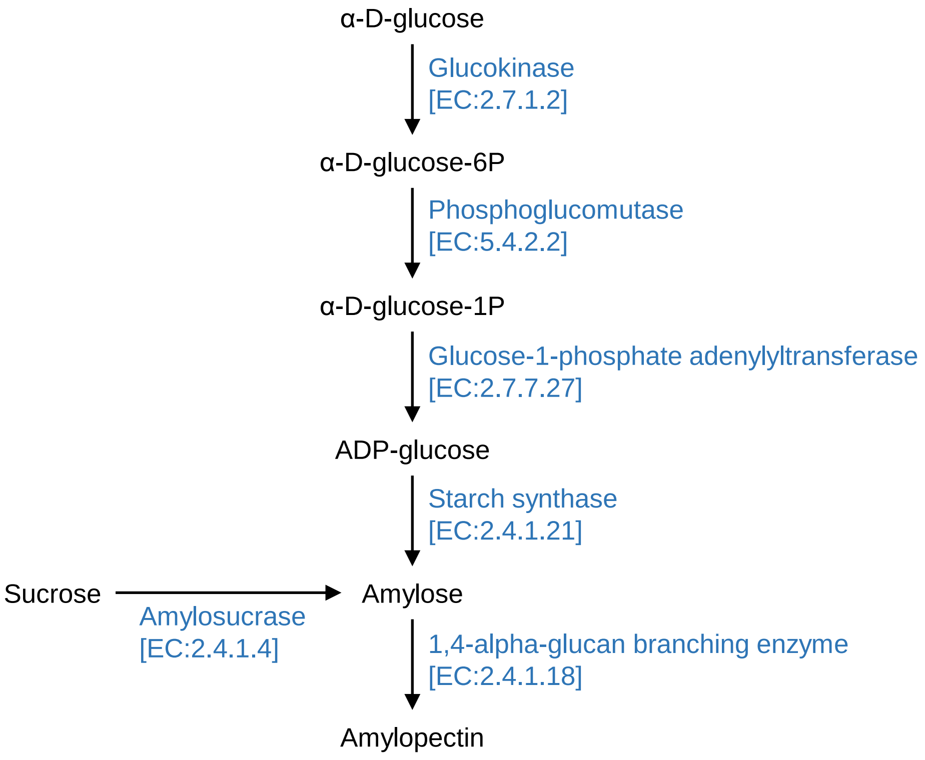

Supplementary Figure 24. KEGG pathway map for starch metabolism in *B. wexlerae*

Supplementary Figure 25

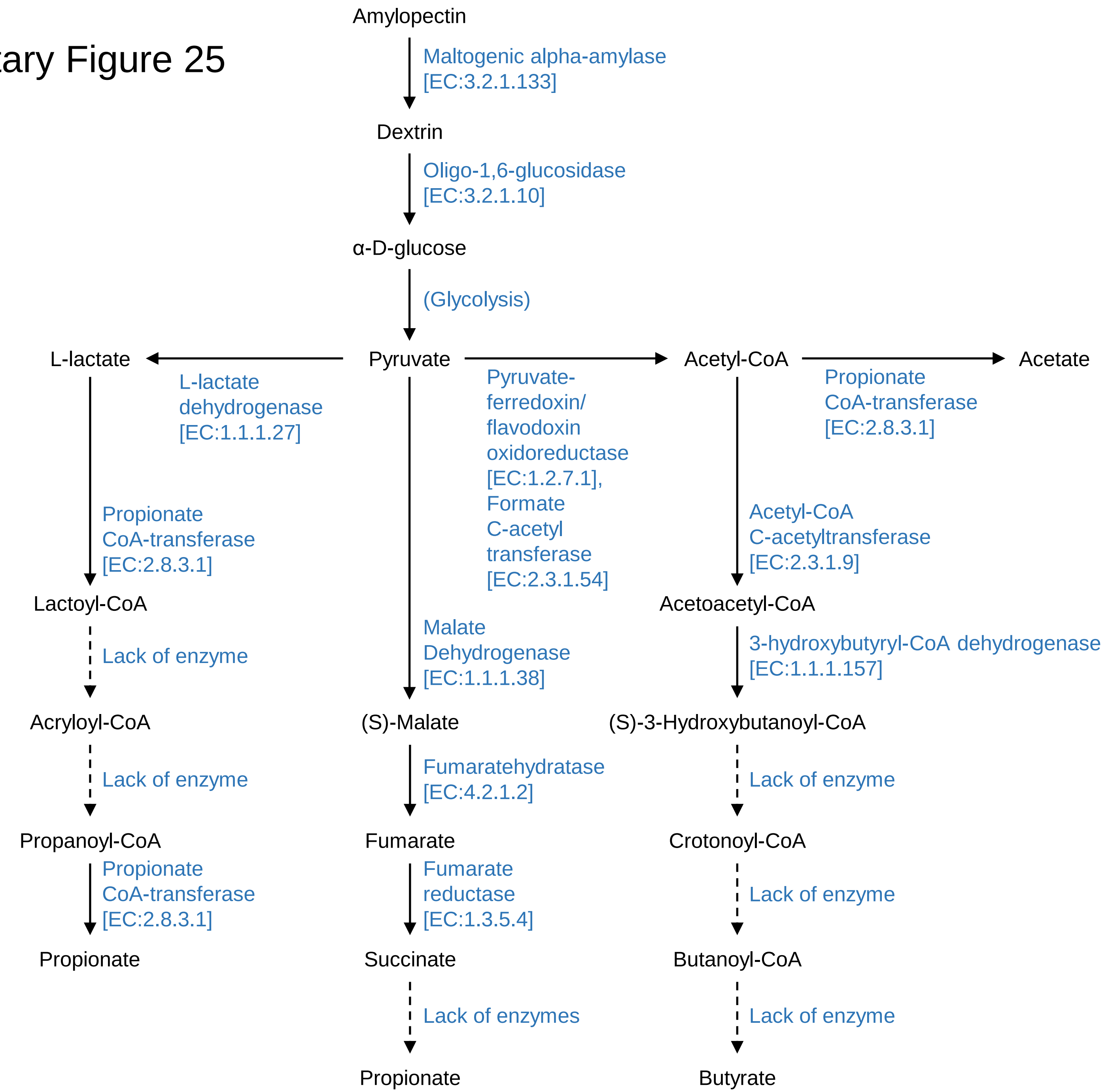

Supplementary Figure 25. KEGG pathway map for carbohydrate metabolism in *B. wexlerae*

Supplementary Figure 26

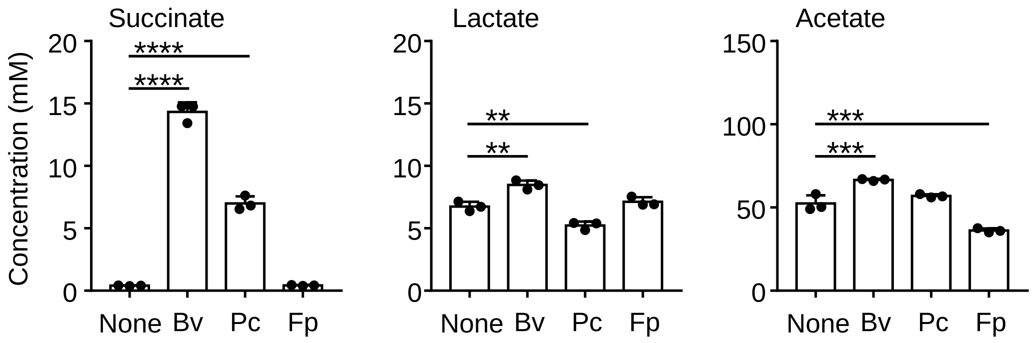

Supplementary Figure 26. The concentrations of succinate, lactate, and acetate in the supernatants from cultures of *Bacteroides vulgatus* (Bv), *Prevotella copri* (Pc), and *Faecalibacterium prausnitzii* (Fp) and in fresh medium (none). Data are representative of 2 independent experiments (n = 3, mean ± 1 SD). Statistical significance was evaluated by using one-way ANOVA; ****P < 0.0001; ***P < 0.001; **P < 0.01;.

Supplementary Figure 27

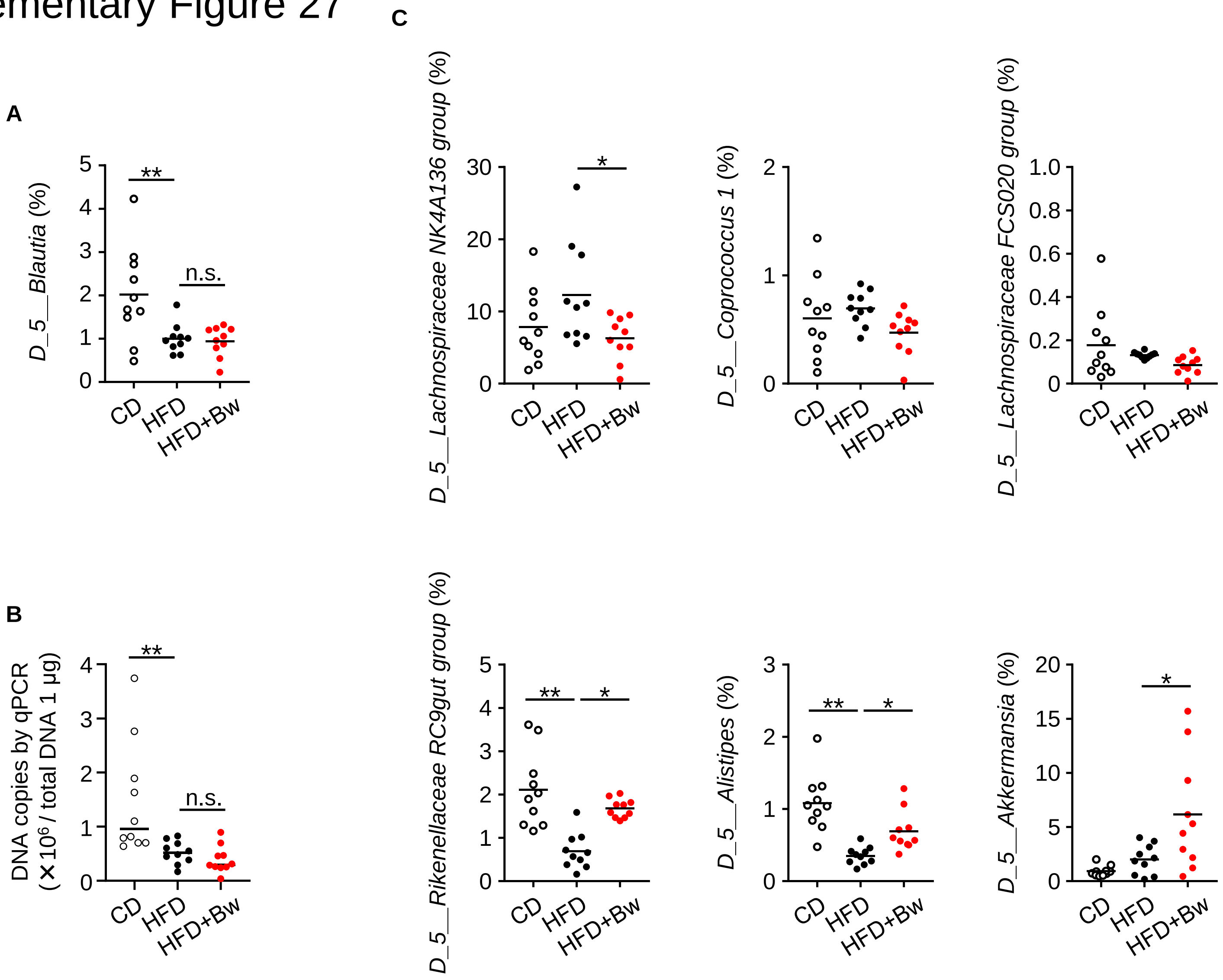

Supplementary Figure 27. Relative abundance of intestinal bacterial genera in mice.
(A) Relative abundance of *Blautia* genus. $**P = 0.0079$; n.s., not significant (one-way ANOVA). (B) DNA copy number of *Blautia* genus by qPCR analysis. $**P = 0.0064$; n.s., not significant (one-way ANOVA). (C) Relative abundance of intestinal bacterial genera ranked according to linear discriminant analysis effect size (Fig. 5F). $*P < 0.05$; $**P < 0.01$ (one-way ANOVA). CD, CD-fed mice; HFD, HFD-fed mice, HFD+Bw, HFD-fed mice supplemented with *B. wexlerae*. Data are combined from 2 independent experiments (n = 10, mean).

Supplementary Table 1. Participant information in discovery cohort

	Total subjects[a]	Non-diabetic subjects (nonDM)	Type 2 diabetes patients (T2DM)
Number	217	147	45
Male/Female	110/107	73/74	23/22
Age (years, mean ± SD)	51.9 ± 13.0 (Range: 30-79)	48.7 ± 11.6 (Range: 30-76)	62.0 ± 12.4[b] (Range: 34-79)
Body mass index (kg/m^2, mean ± SD)	23.0 ± 4.1 (Range: 16.2–39.6)	22.0 ± 2.7 (Range: 17.0–30.6)	26.4 ± 5.9[b] (Range: 16.2–39.6)
Comorbidity			
Hypertension	48 (22%)	19 (13%)	22 (49%)
Dyslipidemia	58 (27%)	27 (18%)	21 (47%)
Laboratory data			
Glucose (mg/dL, mean ± SD)		91.5 ± 8.9[c] (Range: 77-117)	140.5 ± 52.2[d] (Range: 70-323)
HbA1c (%, mean ± SD)		Not tested	7.0 ± 1.2 (Range: 5.6-11.3)
Medication			
Sulfonylureas			10 (22%)
Fast acting insulin secretagogue			4 (9%)
Biguanide			26 (58%)
α-glucosidase inhibitor			4 (9%)
SGLT2 inhibitor			10 (22%)
Incretin-related drugs (DPP-4 inhibitor)			23 (51%)
Insulin preparation			16 (36%)
GLP-1 receptor agonist			3 (7%)

[a] Both T1DM (n=25) and T2DM (n=45) are included in this study because of the recruitment of diabetic patients at the hospital. The analysis of the relationship between BMI (obesity) and intestinal bacteria (Fig. 1A, Supplementary Table 3) was performed by using the data of the 217 total subjects. Because T1DM and T2DM differ in pathogenesis, we chose to focus on T2DM from the viewpoint of diabetes, exclude T1DM, and analyze the relationship between T2DM and intestinal bacteria (Fig. 1B, Supplementary Table 4).

[b] $P < 0.01$ (two-tailed Mann–Whitney *U*-test) between nonDM subjects and T2DM patients

[c] Fasting blood glucose measured at medical examination.

[d] Casual blood glucose measured at hospital.

Supplementary Table 2. Participant information for normal/overweight/obese subgroups of nonDM subjects and T2DM patients in discovery cohort

	Non-diabetic subjects (nonDM)			Type 2 diabetes patients (T2DM)		
	Normal (BMI 18.5–24.9)	Overweight (BMI 25.0–29.9)	Obese (BMI ≥ 30)	Normal (BMI 18.5–24.9)	Overweight (BMI 25.0–29.9)	Obese (BMI ≥ 30)
Number	112	20	2	22	8	14
Male/Female	55/57	16/4	2/0	11/11	4/4	8/6
Age (years, mean ± SD)	49.8 ± 12.3 (Range: 30-76)	47.8 ± 8.2 (Range: 31-68)	43.0 ± 4.2 (Range: 40-46)	65.8 ± 10.1 (Range: 45-78)	65.9 ± 11.8 (Range: 44-79)	53.8 ± 13.1 (Range: 34-74)
Body mass index (kg/m^2, mean ± SD)	21.6 ± 1.7 (Range: 18.6–24.9)	26.2 ± 1.2 (Range: 25.0–28.7)	30.5 ± 0.1 (Range: 30.4–30.6)	22.0 ± 1.7 (Range: 18.9–24.9)	26.3 ± 1.0 (Range: 25.1–27.6)	34.0 ± 2.9 (Range: 30.0–39.6)

Supplementary Table 3. Multiple regression analysis for BMI-related bacteria among 217 total subjects

Genus	Estimate ± SD	T value	*P* value	Mean ± SD (%)	
				Normal (n=148)	Obesity (n=52)
Dorea	0.038 ± 0.010	3.703	0.0003	0.19 ± 0.23	0.29 ± 0.28
Ruminococcaceae.UCG.014	0.014 ± 0.008	1.715	0.0878	0.12 ± 0.05	0.12 ± 0.04
Clostridium.sensu.stricto.1	0.009 ± 0.005	1.679	0.0946	0.11 ± 0.05	0.10 ± 0.05
Lachnospiraceae_uncultured	0.008 ± 0.004	2.080	0.0388	0.51 ± 0.60	0.58 ± 0.72
Dialister	0.007 ± 0.005	1.318	0.1890	0.18 ± 0.42	0.30 ± 0.68
Streptococcus	0.005 ± 0.002	2.981	0.0032	0.55 ± 1.46	0.75 ± 1.36
Barnesiella	0.005 ± 0.003	1.530	0.1277	0.37 ± 0.64	0.52 ± 0.96
Megasphaera	0.002 ± 0.001	1.833	0.0683	0.60 ± 1.73	1.95 ± 3.83
Megamonas	0.002 ± 0.001	2.647	0.0088	0.63 ± 3.07	2.18 ± 5.01
Parabacteroides	0.001 ± 0.001	1.769	0.0784	3.63 ± 5.18	3.45 ± 3.51
Faecalibacterium	-0.001 ± 0.001	-0.966	0.3352	6.41 ± 5.03	4.73 ± 4.73
Blautia	-0.001 ± 0.001	-1.535	0.1263	4.99 ± 4.41	3.31 ± 2.42
Bifidobacterium	-0.001 ± 0.001	-3.279	0.0012	5.35 ± 6.51	2.55 ± 3.28
Lachnospira	-0.005 ± 0.003	-1.927	0.0554	0.70 ± 0.91	0.69 ± 1.10
Butyricicoccus	-0.011 ± 0.006	-1.804	0.0727	0.64 ± 0.48	0.49 ± 0.41
Ruminococcaceae.UCG.005	-0.016 ± 0.006	-2.809	0.0055	0.23 ± 0.75	0.09 ± 0.33

The multiple regression analysis of the relationship between BMI and intestinal bacteria was performed by using the data of the 217 total subjects (Supplementary Table 1).

Supplementary Table 4. Multiple logistic analysis for T2DM-related bacteria among 147 nonDM subjects and 45 T2DM patients

Genus	Estimate ± SD	Z value	*P* value	Mean ± SD (%)	
				nonDM (n=147)	T2DM (n=45)
Flavonifractor	-0.045 ± 0.016	-2.735	0.0062	0.14 ± 0.16	0.20 ± 0.20
Dorea	-0.041 ± 0.015	-2.769	0.0056	0.20 ± 0.22	0.23 ± 0.30
Christensenellaceae.R.7.group	-0.018 ± 0.006	-3.297	0.0010	0.33 ± 1.20	0.40 ± 1.27
Ruminococcus.1	-0.011 ± 0.003	-3.168	0.0015	0.60 ± 1.14	0.75 ± 1.56
Lachnospiraceae_uncultured	-0.010 ± 0.004	-2.317	0.0205	0.51 ± 0.56	0.61 ± 0.80
Ruminococcus.2	-0.010 ± 0.003	-3.360	0.0008	0.25 ± 0.76	0.72 ± 1.20
Streptococcus	-0.005 ± 0.002	-2.429	0.0151	0.52 ± 1.41	0.67 ± 1.42
Prevotella.2	-0.003 ± 0.001	-2.925	0.0034	0.55 ± 3.03	0.97 ± 4.88
Megasphaera	-0.002 ± 0.001	-1.899	0.0575	0.60 ± 1.56	1.62 ± 3.81
Fusobacterium	-0.002 ± 0.001	-1.759	0.0786	0.68 ± 2.96	0.96 ± 3.00
Faecalibacterium	0.002 ± 0.001	2.310	0.0209	6.87 ± 5.08	4.15 ± 4.53
Blautia	0.003 ± 0.001	2.354	0.0186	5.30 ± 4.35	2.65 ± 2.06
Subdoligranulum	0.004 ± 0.001	2.356	0.0185	1.81 ± 2.05	1.34 ± 2.18
Sutterella	0.006 ± 0.002	2.630	0.0085	1.64 ± 1.95	1.24 ± 1.62
Collinsella	0.006 ± 0.002	2.639	0.0083	1.00 ± 1.44	0.71 ± 0.97
Anaerostipes	0.009 ± 0.004	2.337	0.0195	1.36 ± 2.12	0.62 ± 0.65
Butyricicoccus	0.011 ± 0.007	1.656	0.0977	0.68 ± 0.45	0.48 ± 0.45
Alloprevotella	0.012 ± 0.009	1.307	0.1913	0.49 ± 2.49	0.03 ± 0.23
Bilophila	0.014 ± 0.011	1.294	0.1957	0.22 ± 0.26	0.20 ± 0.28
Holdemanella	0.018 ± 0.009	2.080	0.0375	0.16 ± 0.50	0.13 ± 0.40
Prevotellaceae_uncultured	0.087 ± 0.075	1.163	0.2447	0.15 ± 1.31	0.002 ± 0.01
Eubacterium..xylanophilum.group	0.381 ± 0.121	3.152	0.0016	0.04 ± 0.14	0.004 ± 0.02

The multiple logistic analysis of the relationship between T2DM and intestinal bacteria was performed by using the data of the 192 subjects (comprising 147 nonDM subjects and 45 T2DM patients and excluding 25 patients with Type 1 diabetes) (Supplementary Table 1).

Supplementary Table 5. Relationship between *Blautia* and BMI

Blautia (%)	BMI		Odds
	18.5–24.9	≥25.0	
0–2.9	63[a]	32[a]	0.51
3.0–5.9	38[a]	15[a]	0.39
≥6.0	47[a]	5[a]	0.11

[a] Number of participants

Supplementary Table 6. Relationship between *Blautia* and T2DM

Blautia (%)	nonDM	T2DM	Odds
0–2.9	55[a]	33[a]	0.60
3.0–5.9	42[a]	10[a]	0.23
≥6.0	50[a]	2[a]	0.04

[a] Number of participants

Supplementary Table 7. The abundance of *Blautia* genus in human data set randomly adjusted in terms of age and sex.

Data set	Phenotype	Number	Male/ female	Age (years, mean ± SD)	*Blautia* (%, mean ± SD)	*P* value[a]
1	nonDM	53	29/24	58.5 ± 11.2	5.9 ± 3.9	<0.0001
	T2DM	35	19/16	58.8 ± 11.9	2.3 ± 1.7	
2	nonDM	53	29/24	58.4 ± 11.2	5.4 ± 3.8	<0.0001
	T2DM	35	19/16	59.0 ± 12.3	2.5 ± 1.8	
3	nonDM	53	29/24	58.5 ± 11.3	5.1 ± 3.6	0.0001
	T2DM	35	19/16	58.8 ± 11.9	2.7 ± 2.2	
4	nonDM	53	29/24	58.4 ± 11.3	5.6 ± 4.9	<0.0001
	T2DM	35	19/16	58.7 ± 12.0	2.3 ± 1.7	

[a] Comparison of *Blautia* between nonDM subjects and T2DM patients using two-tailed Mann-Whitney *U*-test.

Supplementary Table 8. Participant information in validation cohort

	Total subjects	Normal (BMI 18.5–24.9)	Overweight (BMI 25.0–29.9)	Obese (BMI ≥ 30)
Number	195	132	42	8
Male/Female	114/81	77/55	30/12	3/5
Age (years, mean ± SD)	41.9 ± 11.9 (Range: 21-71)	41.9 ± 11.5 (Range: 23-66)	43.6 ± 12.6 (Range: 22-71)	37.0 ± 13.4 (Range: 21-54)
Body mass index (kg/m^2, mean ± SD)	22.9 ± 3.5 (Range: 16.7–36.6)	21.7 ± 1.8 (Range: 18.6–24.9)	26.5 ± 1.4 (Range: 25.0–29.8)	32.2 ± 2.0 (Range: 30.7–36.6)
Present illness				
Type 2 diabetes	0 (0.0%)			
Hypertension	8 (4.1%)			
Dyslipidemia	21 (10.8%)			
Laboratory data				
Glucose (mg/dL, mean ± SD)	97.9 ± 13.7[a] (Range: 68-228)			
HbA1c (%, mean ± SD)	5.5 ± 0.4 (Range: 4.2-8.9)			

[a] Fasting blood glucose measured at medical examination.
