# Supplementary material for: Oral administration of Blautia wexlerae ameliorates obesity and type 2 diabetes via metabolic remodeling of the gut microbiota
Source: Nat Commun. 2022 Aug 18;13:4477. doi: 10.1038/s41467-022-32015-7 (PMC9388534; doi:10.1038/s41467-022-32015-7)
Supplement: Supplementary file 3 — Reporting Summary [file 41467_2022_32015_MOESM3_ESM.pdf]

nature portfolio

Corresponding author(s): Jun Kunisawa

Last updated by author(s): Jul 8, 2022

Reporting Summary

Nature Portfolio wishes to improve the reproducibility of the work that we publish. This form provides structure for consistency and transparency in reporting. For further information on Nature Portfolio policies, see our Editorial Policies and the Editorial Policy Checklist.

Statistics

For all statistical analyses, confirm that the following items are present in the figure legend, table legend, main text, or Methods section.

n/a	Confirmed	
☐	☒	The exact sample size (*n*) for each experimental group/condition, given as a discrete number and unit of measurement
☐	☒	A statement on whether measurements were taken from distinct samples or whether the same sample was measured repeatedly
☐	☒	The statistical test(s) used AND whether they are one- or two-sided<br>*Only common tests should be described solely by name; describe more complex techniques in the Methods section.*
☐	☒	A description of all covariates tested
☐	☒	A description of any assumptions or corrections, such as tests of normality and adjustment for multiple comparisons
☐	☒	A full description of the statistical parameters including central tendency (e.g. means) or other basic estimates (e.g. regression coefficient) AND variation (e.g. standard deviation) or associated estimates of uncertainty (e.g. confidence intervals)
☐	☒	For null hypothesis testing, the test statistic (e.g. *F*, *t*, *r*) with confidence intervals, effect sizes, degrees of freedom and *P* value noted<br>*Give P values as exact values whenever suitable.*
☒	☐	For Bayesian analysis, information on the choice of priors and Markov chain Monte Carlo settings
☒	☐	For hierarchical and complex designs, identification of the appropriate level for tests and full reporting of outcomes
☐	☒	Estimates of effect sizes (e.g. Cohen's *d*, Pearson's *r*), indicating how they were calculated

Our web collection on statistics for biologists contains articles on many of the points above.

Software and code

Policy information about availability of computer code

Data collection	We do not use previously unreported custom computer code or algorithm.
Data analysis	Our Auto-q Qiime Analysis Automating Script is published in GitHub (https://github.com/Attayeb/auto-q/tree/1.0).<br>We use QIIME pipeline (v1.9.1), R (v3.5.1), and XFe Wave software (v2.3.0).

For manuscripts utilizing custom algorithms or software that are central to the research but not yet described in published literature, software must be made available to editors and reviewers. We strongly encourage code deposition in a community repository (e.g. GitHub). See the Nature Portfolio guidelines for submitting code & software for further information.

Data

Policy information about availability of data

All manuscripts must include a data availability statement. This statement should provide the following information, where applicable:

- Accession codes, unique identifiers, or web links for publicly available datasets
- A description of any restrictions on data availability
- For clinical datasets or third party data, please ensure that the statement adheres to our policy

We use SILVA database v128.
DNA sequencing data generated in this study have been deposited in the DNA Databank of Japan (DDBJ) under the accession numbers DRA010841 [https://ddbj.nig.ac.jp/resource/sra-submission/DRA010841], DRA012134 [https://ddbj.nig.ac.jp/resource/sra-submission/DRA012134], and DRA010839 [https://ddbj.nig.ac.jp/resource/sra-submission/DRA010839].

Field-specific reporting

Please select the one below that is the best fit for your research. If you are not sure, read the appropriate sections before making your selection.

- [x] Life sciences
- [ ] Behavioural & social sciences
- [ ] Ecological, evolutionary & environmental sciences

For a reference copy of the document with all sections, see nature.com/documents/nr-reporting-summary-flat.pdf

Life sciences study design

All studies must disclose on these points even when the disclosure is negative.

Sample size	242 (discovery cohort) and 219 (validation cohort) total possible participants.<br>No statistical method was used to predetermine sample size.
Data exclusions	25 participants who had received antibiotics within the previous 2 weeks (18 subjects), had traveled overseas within the previous month (2 subjects), or had gastrointestinal disease (5 subjects) in the discovery cohort; 24 who had received antibiotics within the previous 2 weeks (10 subjects), had traveled overseas within the previous month (6 subjects), or had gastrointestinal disease (8 subjects) in the validation cohort
Replication	No replication for a cross-sectional study
Randomization	No randomization for a cross-sectional study
Blinding	No blinding for a cross-sectional study

Reporting for specific materials, systems and methods

We require information from authors about some types of materials, experimental systems and methods used in many studies. Here, indicate whether each material, system or method listed is relevant to your study. If you are not sure if a list item applies to your research, read the appropriate section before selecting a response.

Materials & experimental systems

n/a	Involved in the study	
[ ]	[x]	Antibodies
[ ]	[x]	Eukaryotic cell lines
[x]	[ ]	Palaeontology and archaeology
[ ]	[x]	Animals and other organisms
[ ]	[x]	Human research participants
[x]	[ ]	Clinical data
[x]	[ ]	Dual use research of concern

Methods

n/a	Involved in the study	
[x]	[ ]	ChIP-seq
[ ]	[x]	Flow cytometry
[x]	[ ]	MRI-based neuroimaging

Antibodies

Antibodies used	anti-CD16/32 monoclonal antibody (TruStain fcX; Biolegend)<br>BV421–anti-CD45 (Biolegend, clone 30-F11)<br>PE–anti-I-Ab MHC class II (Biolegend, clone AF6-120.1)<br>FITC–anti-CD206 (Biolegend, clone C068C2)<br>PE-Cy7–anti-F4/80 (Biolegend, clone BM8)<br>APC-Cy7–anti-CD11b (Biolegend, clone M1/70)
Validation	https://www.biolegend.com/ja-jp

Eukaryotic cell lines

Policy information about cell lines

Cell line source(s)	3T3L1 cell, JCRB9014
Authentication	The cell line was not authenticated.
Mycoplasma contamination	The cell line was not tested for Mycoplasma contamination.
Commonly misidentified lines (See ICLAC register)	No misidentified lines.

Animals and other organisms

Policy information about studies involving animals; ARRIVE guidelines recommended for reporting animal research

Laboratory animals	Male C57BL/6 mice (age, 4 weeks) were housing in accordance with the guidelines of the Animal Care and Use Committee of the National Institutes of Biomedical Innovation, Health, and Nutrition.
Wild animals	No wild animals were used in the study.
Field-collected samples	The study did not involve samples collected from fields.
Ethics oversight	All animal experiments were approved by the Animal Care and Use Committee of the National Institutes of Biomedical Innovation, Health, and Nutrition (approval no. DS27-48R10).

Note that full information on the approval of the study protocol must also be provided in the manuscript.

Human research participants

Policy information about studies involving human research participants

Population characteristics	217 participants, 110 males/107 females, age 51.9±13.0 (range: 30-79) in the discovery cohort and they contain 147 nondiabetic subjects, 45 type 2 and 25 type 1 diabetic patients; 195 participants, 114 males/81 females, age 41.9±11.9 (range: 21-71) in the validation cohort and they do not contain diabetic patients.
Recruitment	For the discovery cohort, diabetic patients were recruited at Shinnanyo Hospital, Shunan City, Yamaguchi, Japan; nondiabetic adult volunteers (control subjects; e.g., staff members of city offices and chamber of commerce) were recruited at health examination sites in surrounding communities. For a validation cohort, we recruited adult volunteers from among the city office staff of Minamiuonuma City, Niigata, Japan, at health examination sites.<br>Since all subjects participate in the research on their own volition, there is a possibility that people with a high awareness of health, etc. gather.
Ethics oversight	The human research was approved by the Ethics Committee of the National Institutes of Biomedical Innovation, Health, and Nutrition and were conducted in accordance with their guidelines (approval number: 177-08 and Kenei78-06).

Note that full information on the approval of the study protocol must also be provided in the manuscript.

Flow Cytometry

Plots

Confirm that:

- [x] The axis labels state the marker and fluorochrome used (e.g. CD4-FITC).
- [x] The axis scales are clearly visible. Include numbers along axes only for bottom left plot of group (a 'group' is an analysis of identical markers).
- [x] All plots are contour plots with outliers or pseudocolor plots.
- [x] A numerical value for number of cells or percentage (with statistics) is provided.

Methodology

Sample preparation	3T3L1 adipocytes were stained with 100 nM Mitogreen.<br>Cells in the stromal vascular fraction of mouse eAT were stained with an anti-CD16/32 monoclonal antibody (TruStain fcX; Biolegend) to avoid non-specific staining and 7-AAD (Biolegend) to detect dead cells. The cells were further stained with the fluorescently labeled antibodies BV421–anti-CD45 (Biolegend, clone 30-F11), PE–anti-I-Ab MHC class II (Biolegend, clone AF6-120.1), FITC–anti-CD206 (Biolegend, clone C068C2), PE-Cy7–anti-F4/80 (Biolegend, clone BM8), and APC-Cy7–anti-CD11b (Biolegend, clone M1/70).
Instrument	MACSQuant (Miltenyi Biotec) or BD FACSAria III (BD Biosciences)
Software	FlowJo 9.9 (Tree Star, Ashland, Oregon, USA)
Cell population abundance	3T3L1 adipocytes are almost all.<br>The number of macrophages in eAT and percentages of the M1- and M2-like macrophage populations altered in mice because of differences in diet or administration of B. wexlerae.
Gating strategy	The CD45+CD11b+F4/80+ cells were defined as the macrophage population. Among macrophages, the MHC II+/highCD206–/low cells and MHC II+CD206high cells were defined as the M1- and M2-like macrophage populations, respectively.

- [ ] Tick this box to confirm that a figure exemplifying the gating strategy is provided in the Supplementary Information.
